# Insulin signaling shapes fractal scaling of *C. elegans* behavior

**DOI:** 10.1038/s41598-022-13022-6

**Published:** 2022-06-21

**Authors:** Yukinobu Arata, Itsuki Shiga, Yusaku Ikeda, Peter Jurica, Hiroshi Kimura, Ken Kiyono, Yasushi Sako

**Affiliations:** 1 Cellular Informatics Laboratory, Cluster for Pioneering Research (CPR), RIKEN, 2-1 Hirosawa, Wako, Saitama 351-0198 Japan; 2 Graduate School of Engineering Science, Osaka University, 1-3 Machikaneyama-cho, Toyonaka, Osaka 560-8531 Japan; 3 Department of Mechanical Engineering, School of Engineering, Tokai University, 4-1-1 Kitakaname, Hiratsuka, Kanagawa 259-1292 Japan

**Keywords:** Biophysics, Neuroscience, Feeding behaviour, Molecular neuroscience

## Abstract

Fractal scaling in animal behavioral activity, where similar temporal patterns appear repeatedly over a series of magnifications among time scales, governs the complex behavior of various animal species and, in humans, can be altered by neurodegenerative diseases and aging. However, the mechanism underlying fractal scaling remains unknown. Here, we cultured *C. elegans* in a microfluidic device for 3 days and analyzed temporal patterns of *C. elegans* activity by fractal analyses. The residence-time distribution of *C. elegans* behaviors shared a common feature with those of human and mice. Specifically, the residence-time power-law distribution of the active state changed to an exponential-like decline at a longer time scale, whereas the inactive state followed a power-law distribution. An exponential-like decline appeared with nutrient supply in wild-type animals, whereas this decline disappeared in insulin-signaling-defective *daf-2* and *daf-16* mutants. The absolute value of the power-law exponent of the inactive state distribution increased with nutrient supply in wild-type animals, whereas the value decreased in *daf-2* and *daf-16* mutants. We conclude that insulin signaling differentially affects mechanisms that determine the residence time in active and inactive states in *C. elegans* behavior. In humans, diabetes mellitus, which is caused by defects in insulin signaling, is associated with mood disorders that affect daily behavioral activities. We hypothesize that comorbid behavioral defects in patients with diabetes may be attributed to altered fractal scaling of human behavior.

## Introduction

In humans, ordinary daily activities^1^ and social activities (e.g., communication, entertainment, and work^2,3^) tend to occur consecutively as a burst, and then suddenly cease on the time scale from several seconds to hours (for daily activities) or to several days (for social activities). Such episodic bouts of behavior have also been observed in other vertebrates (mice^4^) and invertebrates (*C. elegans*^5^, flies^6^, and ants^7^). Activity time series of episodic behavioral bouts have non-periodic and intermittent patterns that appear repeatedly across a broad range of time scales. Self-similar geometrical patterns across time scales are called fractal patterns; therefore, activity time series of animal behavior are characterized by fractal geometry. Neurodegenerative disorders (e.g., Alzheimer’s and Parkinson’s diseases) or aging have been shown to alter the fractal scaling of various human behaviors^8–11^. These findings suggest that fractal scaling of animal behavior is regulated by neurophysiological mechanisms, which may be conserved among various animal species.

Daily and social activities are affected by a broad range of neurophysiological states in the human brain. Among them, mood, an unconscious disposition to respond emotionally to objects or events encountered in life^12^, and a reward evaluation for each object or event^13^ are thought to play important roles. Insulin signaling has been shown to affect mood and the reward system in mouse and human brains^14^. Mice with a brain neuron-specific knockout of the insulin receptor gene (NIRKO mice) did not show defects in neuron proliferation or death during brain development; however, they did show age-related anxiety and depressive-like behaviors^15^. In humans, nasal administration of insulin, which is mainly incorporated and functions in the brain without affecting blood glucose concentration in the periphery^16^, improved mood in both healthy individuals and patients with diabetes^17^. These results suggest that insulin signaling functions in mood control in the mouse and human brain. Insulin signaling, which has evolved in relation to the mood and reward systems in brain in higher animals, modulates the relation between olfactory stimuli and behavior in nematodes and flies^14^. Thus, insulin signaling is an evolutionarily conserved signaling system that coordinates external stimulation and animal behavior via neurosensory modulation. However, how insulin signaling affects the fractal scaling of animal behavior remains uninvestigated.

In the present study, we applied a genetic analysis of the fractal scaling of animal behavior by studying *C. elegans* behavior. Alternating switching between an actively moving state (“active state”) and an inactive state in episodic behavior is a common feature of various animal species. Therefore, we dissected the fractal scaling of *C. elegans* behavior on a two-state transition model^5^. Generally, kinetics that governs the state transition can be inferred from statistical properties, such as the frequency distribution and temporal correlation of experimentally measured residence times in each state. Inferred kinetics provides insights into the underlying mechanisms that drive the state transition. Through longitudinal videorecording of *C. elegans* swimming behavior, we found that state transitions between active and inactive states in *C. elegans* episodic behavior are driven by kinetics that determines residence times by following frequency distributions and temporal correlations with fractal properties. Therefore, we refer to the kinetics as “fractal kinetics”^5^.

In this study, we extended the function of the microfluidic device for culturing *C. elegans* with food bacteria. Specifically, we examined wild-type *C. elegans* cultured with or without nutrient source (i.e., *E. coli* and glucose), as well as *daf-2* and *daf-16* nematode mutant strains cultured with nutrient source. The mutant strains have mutations in the insulin-like receptor gene (*daf-2*) and in the downstream forkhead transcription factor gene (*daf-16*)^18^. Our observations revealed that the fractal kinetics of *C. elegans* behavior is regulated by insulin signaling. Based on recent neuronal network modelling and molecular biological studies, we discuss the possibility that insulin signaling regulates neural activity in the brain to modulate fractal scaling of *C. elegans* behavior. We also discuss the applicability of this mechanism for mood disorders that are comorbid with diabetes mellitus in humans. We propose that fractal behavioral analysis can provide a more integrated clinical view of psychiatric symptoms in patients with diabetes, which may contribute to the development of new diagnostic indices and improvement of clinical treatment.

## Results

### Residence-time power-law distributions in the active and inactive states in *C. elegans* episodic behavior

**Figure 1:**
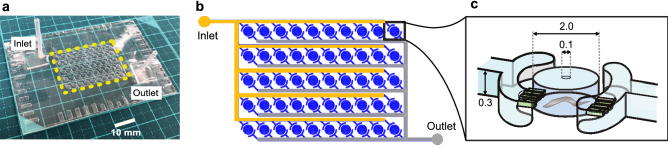
WormFloII microfluidic device for culturing *C. elegans* with food bacteria in biochemical isolation. (**a**) WormFloII photo. Inlet and outlet portals for liquid media are shown. Yellow dashed line outlines 50 chambers for individually culturing *C. elegans*. (**b**) WormFloII schematic. Each chamber is directly connected to supply (orange) and drain (gray) channels to achieve biochemically independent environments. (**c**) Chamber schematic. Chambers are caged with junctional micro-slit channels (50-μm width and height). Animals are introduced from 0.1-mm hole at chamber roof. Hole is shielded with PDMS sheet before supplying liquid media.

To study the effects of diet on the fractal scaling of *C. elegans* behavior, we constructed a new microfluidic device composed of an array of 50 chambers for culturing individual animals by perfusing M9 buffer containing food bacteria (WormFloII, Fig. 1). We recorded *C. elegans* swimming under controlled chemical, temperature, and light intensity conditions at 20 frames per second (fps) for 3 days^5^. By analyzing recorded movies using an image-processing program^5^, we obtained time series of behavioral activity with 10^5^ time points (Extended Data Fig. 1a). In the activity time series, we confirmed that fed wild-type animals cultured on the device showed repeated active and inactive episodes (Extended Data Fig. 1b) and a high-low activity pattern that appeared repeatedly over a series of magnifications among time scales (Extended Data Fig. 1c–e). A similar high-low activity pattern was previously observed in *C. elegans* cultured in liquid^5^ and solid agar medium^19^. These findings indicate that our culture system allowed us to observe the physiological behavior of *C. elegans*.

Due to the sudden switching of behavioral states in an episodic manner, we separately analyzed the statistical properties of residence times in the active and inactive states. The residence time in the active state (how long animals were moving) and the residence time in the inactive state (how long animals maintained the same posture or remained less active) were measured alternatingly along an activity time series. In step 1, we obtained the residence time for the first round of the active state and the residence time for the first round of the inactive state. Similarly, in step 2, we obtained the residence time for the second round of the active state and the residence time for the second round of the inactive state. This measurement process continued such that in step *n*, we obtained the residence times for the *n*th round of each of the active and inactive states, until the entire activity time series for 3 days was analyzed.

**Figure 2:**
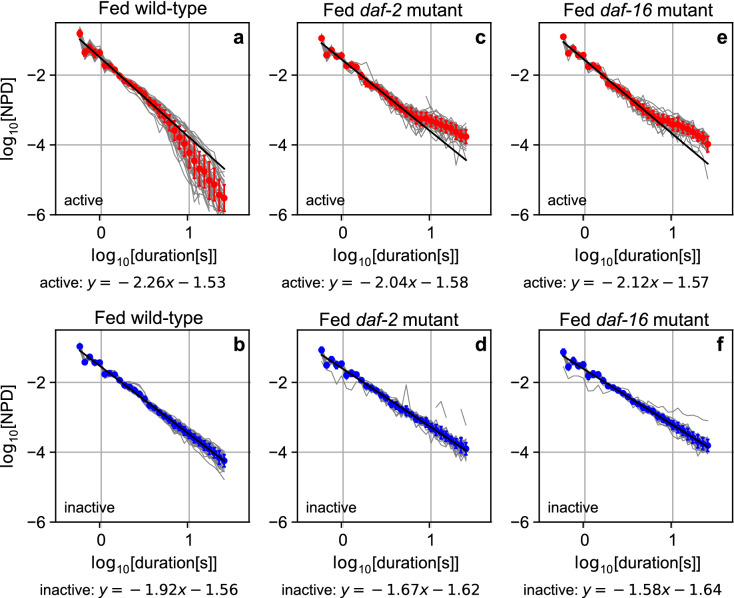
Power-law residence-time distributions of behavioral states of fed *C. elegans* Averaged normalized probability density distributions of active (red) and inactive states (blue) of fed wild-type (**a**,**b**), *daf-2* (**c**,**d**), and *daf-16* (**e**,**f**) animals among individual animals (grey), in log–log plot. Error bars represent standard deviations. Distributions for inactive state (**b**,**d**,**f**) were fit with linear function in the entire range in the residence time from − 0.5 to 1.5 on x-axis (black line). To visually track the presence of the exponential-like tail in the residence distributions for the active state (**a**,**c**,**e**), the distributions were fit with a linear function in the shorter range in the residence time from − 0.5 to 0.8 and were extrapolated to 1.5 on x-axis (black line).

Residence-times in the active or inactive state were plotted across the round, as “duration (*vs.*) round series” (DRS) (Extended Data Fig. 1f,g) analogous to “activity (*vs.*) time series”. DRS in fed wild-type animals revealed that residence times in the active state varied from sub-seconds to 10 s, whereas residence times in the inactive state varied from sub-seconds to 100 s (Extended Data Fig. 1f,g). In the inactive state, residence times followed a power-law distribution in the range of sub-seconds to > 10 s (Fig. 2b). In the active state, residence times followed a power-law distribution in a shorter range, from sub-seconds to < 10 s (Fig. 2a). This power-law distribution indicates that the residence time appearance frequency decreased on the time scale in a certain ratio across a broad range of residence times. In other words, the appearance frequency decreased in a self-similar manner, which is indicative of fractal scaling in the residence time. Interestingly, at a longer time scale, the frequency distribution of residence time in the active state showed a faster decline than the power-law distribution. Such a convex decline in the log–log plot is seen in an exponential distribution. A similar combination of frequency distributions with and without the exponential-like decline in active and inactive states, respectively, was reported in mice and humans^1,4^. Thus, the frequency distribution of *C. elegans* episodic swimming has a common scaling property to these vertebrates.

Next, we studied the behavioral activity of *C. elegans* that had been cultured in M9 buffer alone (starved wild-type animals) or cultured with 1 g/L glucose (glucose-fed wild-type animals) (Extended Data Fig. 2). The residence-time distribution of starved wild-type animals did not show a detectable exponential-like decline in either the active or inactive state (Extended Data Fig. 3a,b)^5^. In glucose-fed wild-type animals, the exponential-like decline was restored in the active state (Extended Data Fig. 3c,d), raising the possibility that insulin signaling is involved in regulation of fractal scaling of *C. elegans* behavior. To test this possibility, we studied the *daf-2* (Extended Data Fig. 1h–n) and *daf-16* (Extended Data Fig. 1t–u) mutant animals. In both cases, the insulin-signaling mutants showed increased frequency of the long-lasting active state compared to fed wild-type animals, such that the exponential-like decline at the longer time scale disappeared in the active state (Fig. 2c,e). The tail distributions in the average residence time distributions among individual animals (red lines in Fig. 2 and Extended Data Fig. 3) showed the same trend as the tail distributions in each animal (grey lines in Fig. 2 and Extended Data Fig. 3), indicating that the insulin signaling-dependent change in the tail distribution was not due to under-sampling. The absolute value in the power-law exponent for the inactive state in fed wild-type animals decreased in fed *daf-2* and *daf-16* mutants (Fig. 2b,d,f, and $p<0.05$, Extended Data Figs. 4). The absolute value in the power-law exponent for the inactive state in starved wild-type animals was restored in glucose-fed wild-type animals (Extended Data Figs. 3b,d, and $p<0.05$, Extended Data Figs. 4). Therefore, we conclude that the mechanisms to determine residence-time distributions in the active and the inactive state are differentially controlled by insulin signaling.

### Long-range correlation in duration round series of the active and inactive states in C. *elegans* episodic behaviors

To study whether the residence time in the active or inactive state in a round has a prolonged effect across subsequent rounds in the DRS, we focused on the autocorrelation of DRSs. When the autocorrelation of a one-dimensional data series declines with time lag $\tau$ in a power-law manner ($C\left( \tau \right) \sim \tau^{ - \gamma }$), such an autocorrelation is referred to as “long-range correlation,” due to the long tail in the power-law distribution. A power-law distribution of autocorrelation indicates that autocorrelation declines in a certain ratio across a broad range of time-lags, i.e., autocorrelation declines in a self-similar manner, which is indicative of fractal scaling across the round of residence times.

To study the long-range correlation in fractal scaling of *C. elegans* behavior, we employed higher-order detrending moving-average analysis (DMA)^20^. In DMA and its two-variable extension, detrending moving-average cross-correlation analysis (DMCA), when the fluctuation functions ($F\left( s \right)$ or $F^{{\left( {1,2} \right)}} \left( s \right)$, Eqs. (1), (2)) follow a power law with scale ($s$)$(F\left( s \right)\sim s^{\alpha }$ or $F^{{\left( {1,2} \right)}} \left( s \right)\sim s^{\alpha }$), $\alpha$ corresponds to the Hurst exponent $\left( H \right)$^20,21^. $H$ obtained by DMA and DMCA has a direct mathematical link with other conventional indices for long-range correlation: i.e., the scaling exponent $\gamma$ in autocorrelation ($\gamma = 2 - 2\alpha ,$ for $0 < \gamma < 1$) and the scaling exponent $\beta$ in power spectral density $P\left( f \right)\sim f^{ - \beta }$, where $f$ is the frequency ($\beta = 2\alpha - 1$, for $\beta > - 1$)^22^. Compared to conventional algorithms, DMA has several advantages for estimating long-range correlation for scaling exponent $\gamma$ or $\beta$, due to the availability of a fast algorithm and improved trend removal process^20^. When $H = 0.5$, the time series had no temporal correlation (i.e., like white noise), whereas when $0.5 < H < 1$, the time series had a long-range correlation. Although long-range correlation of the time series cannot be simply extended to $H > 1$ due to $0 < \gamma < 1$, fractal scaling of the time series can be characterized by $H > 1$. When $H$ is larger ($H > 0.5$), there is a stronger tendency for values in the time series to continuously increase or decrease^5,20,23^. Therefore, in our study, we classified fractal scaling of the time series as “no memory” at $H = 0.5$, “weak fractal memory” for $0.5 < H < 1$, and “strong fractal memory” at $H > 1$. Note that we used $H$ of the integrated time series to characterize fractal scaling of the original time series, by following a standard algorithm of DMA (Methods).

**Figure 3:**
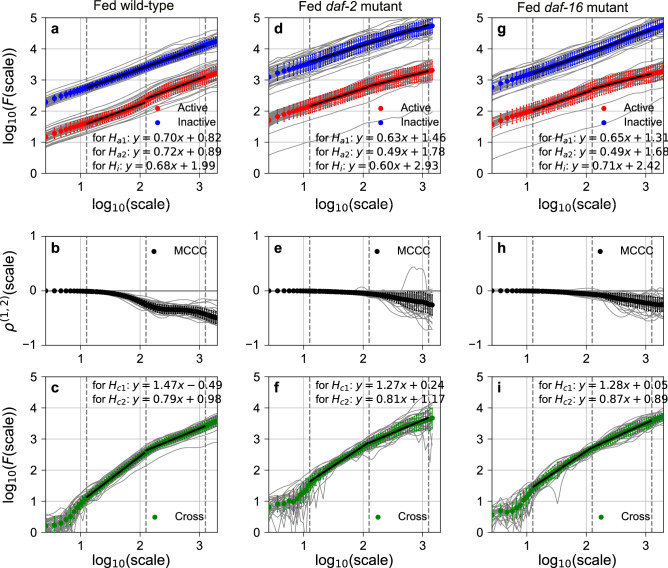
Long-range auto-/cross-correlations and multiscale cross-correlation coefficients in fed *C. elegans* behavior. Averaged noise function ${\varvec{F}}\left( {\varvec{s}} \right)$ of active (red) and averaged cross-noise function ${\varvec{F}}^{{\left( {1,2} \right)}} \left( {\varvec{s}} \right)$ (green) among individual animals (grey) were fit with linear function from 1.1 to 2.1 and from 2.1 to 3.1 across scale $\left( {\varvec{s}} \right)$ at shorter and longer round scale for fed wild-type (**a**,**c**), *daf-2* (**d**,**f**), and *daf-16* (**g**,**i**), respectively. Averaged noise function ${\varvec{F}}\left( {\varvec{s}} \right)$ of inactive (blue) DRSs was fit from 1.1 to 3.1. Averaged multiscale cross-correlation coefficient (MCCC; black) among individual animals (grey) were plotted against scale $\left( {\varvec{s}} \right)$ for fed wild-type (**b**), *daf-2* (**e**), and *daf-16* (**h**). Error bars are standard deviation.

The Hurst exponent of DRS of the active state (active DRS) at shorter round scale ($< 100$ rounds, $H_{a1} = 0.70$) and that at longer round scale ($> 100$ rounds, $H_{a2} = 0.72$) (Fig. 3a), and the Hurst exponent of DRS of the inactive state (inactive DRS) ($H_{i} = 0.68$) (Fig. 3a) indicate that the active and inactive DRSs have weak fractal memories, consistent with our previous study^5^. We did not find strong evidence for insulin signaling-dependent control of the mechanism to determine temporal correlations in active and inactive DRSs (Fig. 3d,g, Extended Data Figs. 5, 6a–f and Supplementary Discussion).

### Cross-correlation between duration round series of the active and inactive states in *C. elegans* episodic behavior

To study the relation between active and inactive DRSs, we estimated the cross-correlation coefficients between two DRSs at various temporal scales (multiscale cross-correlation coefficient, $\rho^{{\left( {1,2} \right)}} \left( s \right)$, Eq. (3)). Fed wild-type animals showed a remarkable negative correlation at longer round scales (Fig. 3b, Extended Data Fig. 7). The negative correlation at longer round scale ($log_{10} \left( s \right) = 2.5$) in fed wild-type animals ($\rho_{2.5} = - 0.35$) was significantly weakened in insulin-signaling mutant animals ($\rho_{2.5} = - 0.10$ and $\rho_{2.5} = - 0.10$ in fed *daf-2* and fed *daf-16* mutants, respectively, Fig. 3e,h, $p < 0.05$, Extended Data Fig. 7). The negative correlation in starved wild-type animals ($\rho_{2.5} = - 0.09$) was restored in glucose-fed wild-type animals ($\rho_{2.5} = - 0.13$, Extended Data Fig. 5b,e, $p < 0.05$, Extended Data Fig. 7). These results indicate that there is a lateral linking mechanism between the two fractal kinetics (to determine active and inactive DRSs) at longer round scale, whose switch is modulated by insulin signaling.

Next, to study the *long-range* cross-correlation between active and inactive DRSs, we employed DMCA. In fed wild-type animals, Hurst exponents of a cross-correlated component between active and inactive DRSs at a shorter round scale ($H_{c1} = 1.47$) and at a longer round scale ($H_{c2} = 0.79$) (Fig. 3c) indicate that active and inactive DRSs contain a cross-correlated fractal component with strong fractal memory at a shorter round scale and weak fractal memory at a longer round scale. At a shorter round scale, $H_{c1}$ in fed wild-type animals ($1.47$) was decreased in insulin-signaling mutants ($1.27$ and $1.28$ in fed *daf-2* and fed *daf-16* mutants, respectively; Fig. 3f,i, $p < 0.05,$ Extended Data Fig. 6g,h). Additionally, $H_{c1}$ in starved wild-type animals ($1.00$) was restored in glucose-fed wild-type animals ($1.20$) (Extended Data Fig. 5c,f, $p < 0.05,$ Extended Data Fig. 6g,h). These results indicate that the strength of fractal memory in the cross-correlated component, unlike the fractal memories of active and inactive DRSs, is controlled by insulin signaling.

To our knowledge, there is no simple model to increase the strength of fractal memory by coupling simple models that generate time series with a weaker fractal memory^24^. Therefore, we consider that a DRS with a strong fractal memory generated by an upstream fractal kinetics is provided to both the active and inactive DRSs as a pseudo-cross-correlated component via a vertical interaction mechanism. On the other hand, at longer round scale, $H_{c2}$ in fed wild-type animals ($0.79$, Fig. 3c) was comparable to the fractal memory of the active or inactive DRSs in fed wild-type animals (0.72 and 0.68, Fig. 3a). These values did not change significantly in insulin-signaling mutants (0.81 and 0.87 in fed *daf-2* and fed *daf-16* mutants, respectively; Fig. 3f,i, Extended Data Fig. 6i,j). Due to the comparable strength of the fractal memory in $H_{c2}$ compared to those in $H_{a2}$ and $H_{i}$, the presence of an upstream fractal kinetics for a cross-correlated component remained unclear. It is possible that the DRS generated by fractal kinetics for an active or inactive DRS was provided to the other DRS as a cross-correlated component via a lateral linking mechanism, which may be the same as the lateral linking mechanism found by the multiscale cross-correlation coefficient above. How insulin signaling-dependent behavioral control detected by DRS-based analyses alters the temporal activity patterns of *C. elegans* behavior is discussed in the Supplementary Discussion (Extended Data Figs. 8, 9).

## Discussion

### Dissection of fractal scaling of *C. elegans* behavior in a two-state transition model

Generally speaking, the statistical properties of the residence time in each state represent the kinetics governing the transition between the states. We found that the residence time distributions in animal behavioral states and the temporal correlation of the appearance of residence times along the round followed a self-similar fractal distribution. Due to the sudden switching of the behavioral states and the fractal nature of the statistical properties of the residence time appearance, we dissected the fractal behavioral scaling of *C. elegans* behavior using a two-state transition model with “fractal kinetics”^5^. The transition from the active to inactive state is driven by kinetics that determine residence time in the active state by following power-law and exponential-like distributions at shorter and longer time scales (Fig. 2a). The temporal correlation of residence times across the round is determined by following weak fractal memories with distinct strengths at shorter and longer round scales ($H_{a1}$ and $H_{a2}$; Fig. 3a). We refer to such kinetics as Fractal Kinetics A1 and A2, respectively. The temporal correlation determined by Fractal Kinetics A1 is affected by Fractal Kinetics C, which determines the temporal correlation by following strong fractal memory, via the vertical linking mechanism ($H_{c1} ;$ Fig. 3c). The temporal correlation determined by Fractal Kinetics A2 is affected by Fractal Kinetics I via the lateral linking mechanism ($\rho_{2.5}$; Fig. 3b). On the other hand, the state transition from the inactive to active state is driven by kinetics that determines the residence time in the inactive state by following power-law distributions (Fig. 2b). The temporal correlation of residence times across the round is determined by following weak fractal memory ($H_{i}$; Fig. 3a). We refer to such kinetics as Fractal Kinetics I. The temporal correlation determined by Fractal Kinetics I is affected by Fractal Kinetics C via the vertical linking mechanism at shorter round scale ($H_{c1} ;$ Fig. 3c) and by Fractal Kinetics A2 via the lateral linking mechanism at longer round scale ($\rho_{2.5}$; Fig. 3b). Insulin signaling modulates the mechanism for determining the tail of the residence-time distribution in Fractal Kinetics A1 and A2 (Fig. 2a,c,e), and the mechanism for determining fractal memory in Fractal Kinetics C ($H_{c1} ;$ Fig. 3c,f,i) and the switch for the lateral linking mechanism between Fractal Kinetics A2 and I ($\rho_{2.5}$; Fig. 3b,e,h). Insulin signaling also targets the power law exponents of residence-time distribution in Fractal Kinetics I to shape fractal scaling of *C. elegans* behavior. Our finding indicates that animal behavior, which is regulated by complex physiological dynamics in a neuro-muscular network, can be described based on such a simple mathematical/physical framework. Our measurement-based modeling on animal behavior enables the physical basis of animal behavior to be studied by genetics approach.

The generator of fractal kinetics may reside in the neural network in *C. elegans* “brains”. Power-law distributions have been observed in the size and duration of the sequential firing of neurons, called a “neuronal avalanche”, on cultured rat brain slices^25^ and in cat, monkey, and human brains in vitro and in vivo^26^. Exponents of the power-law distribution of neuronal avalanche *duration* are typically distributed around − 2^25,27–29^, which approximately coincides with the power-law exponents of duration of behavioral states in locomotor activity in mouse^4^ and human^1–3^. Neuronal avalanche dynamics in the brain varies in correlation with power-law dynamics in behavioral fractal scaling among individuals on each resting or task-performing behavioral state^27^. Based on these observations, it has been proposed that brain neuronal avalanche dynamics underlies the power-law distribution in behavioral fractal kinetics^1,4,30^. Moreover, the power law dynamics of neuronal avalanche has been reproduced by various theoretical neural network (NN) models^26^, including: stochastic NN models based on second-order phase transition (referred to as “criticality”)^31^, a deterministic model based on a feed forward-type NN model^32^, and another deterministic model at the “edge of chaos”^33^, indicating that power law neural avalanche dynamics is due to the collective dynamics of neuron in the brain. Together, these studies suggest that animal fractal behavioral scaling is derived from collective dynamics of neurons in the brain. In *C. elegans*, we found that the residence-time power-law distributions in the active and inactive states had the same distribution shapes and − 2 power law exponents (Extended Data Fig. 4) as were previously found in human and mice^1,4^. Using activity time series of neurons obtained from whole-brain calcium imaging with cellular resolution, researchers have discussed the possibility that the *C. elegans* brain works at the criticality^34^. Thus, *C. elegans* behavioral fractal kinetics may be derived from a conserved principle of the collective neural dynamics of neurons among *C. elegans*, mouse and human.

### Insulin signaling may regulate fractal kinetics by controlling neural activity in brain

**Figure 4:**
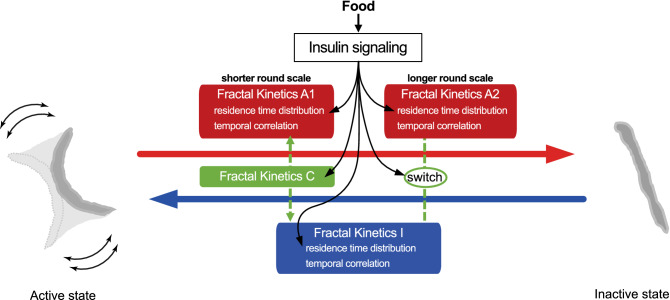
Two-state behavioral transition model. State transition from the active to inactive state is driven by Fractal Kinetics A1 at shorter round scale (which is affected by Fractal Kinetics C) and is driven by fractal Kinetics A2 at longer round scale (which is affected by Fractal Kinetics I). State transition from the inactive to active state is driven by Fractal Kinetics I, which is affected by Fractal Kinetics C at the shorter round scale, and is affected by Fractal Kinetics A2 at the longer round scale. Insulin signaling targets Fractal Kinetics A1, A2, C, and the lateral linking mechanism.

Because DAF-2 negatively regulates DAF-16 activity in the insulin signaling pathway, *daf-2* and *daf-16* mutants are known to exhibit biologically opposite phenotypes^35^. For example, the *daf-2* mutant has a long lifespan, whereas the *daf-16* mutant has a short lifespan^18^. Another example regards entry into the dauer diapause state, with dauer-constitutive (Daf-c) vs. dauer-formation defective (Daf-d) phenotypes for the *daf-2* and *daf-16* mutants, respectively^35^. Unexpectedly, we found that most of the Fractal Kinetics parameters summarized in Fig. 4 showed similar changes in both mutants. Among the indicators in our fractal analysis, only the fractal memory in inactive DRS tended to be opposite between *daf-2* and *daf-16* mutants (Extended Fig. 6e), although the effect was too small to conclude that this parameter is under the control of insulin signaling. The similar change in Fractal Kinetics in *daf-2* and *daf-16* mutants may occur because the insulin signaling-dependent control in the behavioral Fractal Kinetics requires ON–OFF dynamics rather than the ON- or OFF-state in insulin signaling. According to autonomous switching between active and inactive states in *C. elegans* episodic behavior, the rate of nutrient intake may change. Thus, insulin signaling may repeatedly switch between the ON and OFF states, which may cause a complex regulation of insulin signaling on episodic behavior. Even in *C. elegans*, insulin signaling is known to function in various organs^35–38^. The mechanisms of insulin signaling for modulating fractal behavioral scaling need to be studied not only by fractal behavioral analysis in starved *daf-2* and *daf-16* mutants, but also through genetic and molecular biological experiments for insulin signaling at cell or organ resolution.

In seeking to understand the regulation of Fractal Kinetics by insulin signaling, *C. elegans* brain offers an attractive model. Previous NN model analyses and subsequent experiments consistently explained the altered power-law residence-time distribution in fractal kinetics A1/A2 in insulin-signaling mutants (Fig. 2c,e). In previous stochastic and deterministic NN models, the power-law distribution of neuronal avalanche duration changes to an exponential-like decline at longer time scale, as we found in fed wild-type animals (Fig. 2a), when the maximum number of neurons in the model is reduced^31^ or neural activity is suppressed^33^. These models suggest that negative interventions on propagation of neuronal firing in the network cause the exponential-like decline. This prediction has been validated in experiments using cultured rat brain slices^39^. When a brain slice was cultured with an inhibitory neuron antagonist (i.e., picrotoxin), the average neuronal avalanche duration was elongated, such that the exponential-like decline disappeared from the frequency distribution observed in a brain slice cultured without the antagonist. Interestingly, with an inhibitory neuron antagonist, the frequency distribution at the longer time scale was beyond the power-law distribution, similar to what we observed in fed *daf-2* and fed *daf-16* animals (Fig. 2c,e). These theoretical and experimental evidences suggest that the exponential-like decline in the residence-time distribution observed in *C. elegans* and other animals^2,4,9,40^ may be derived from some negative effects on brain neural activity by insulin signaling.

Previous molecular biological analyses have shown that in mammals, insulin acts as a neuropeptide to activate the GABA inhibitory ganglia in amygdala^41^, a key brain region connecting emotion/mood with food intake, providing a negative effect on neural activity. In *C. elegans*, GABA inhibitory D type-motor neurons (D-MNs) are involved in behavioral threat-reward decision making, although the involvement of insulin signaling in this process has not been studied in *C. elegans*^42^. The mechanism to determine the power law and exponential-like distribution of residence time in the active state shared in Fractal Kinetics A1 and A2 may be generated from the specific neuronal network containing D-MNs in *C. elegans,* as an analogy to the GABA inhibitory ganglia in amygdala in mammalians. Based on our findings, together with previous theoretical and experimental studies, we propose the hypothesis that insulin signaling activates GABA inhibitory neurons and alters the power-law neuronal avalanche distribution to be exponential-like, thereby changing the accompanying power-law residence-time distribution to be exponential-like in *C. elegans* behavior. With regard to Fractal Kinetics I and C, how they are generated and how their interactions in the kinetic regulatory pathway (Fig. 4) are achieved in the *C. elegans* brain, remain unknown. Considering the correlation of power-law exponents of animal behavior and neuronal avalanche and the evolutionary conservation of insulin signaling, the insulin-dependent control of fractal kinetics in the kinetic regulatory pathway (Fig. 4) may be conserved in fractal behavioral scaling in other animals.

### Human behavioral analysis for diabetes-associated psychiatric symptoms and the possibility of developing new diagnoses based on fractal statistical indices

In humans, diabetes mellitus is associated with mood disorders, such as depression, bipolar disorder, and generalized anxiety disorder^12^, which affect daily activities, including food intake, sleep, and communication, and social activities. These activities occur at different time scales. Our *C. elegans* fractal behavioral analysis raises the possibility that daily behavioral disorders in patients with diabetes at different time scales may be attributed to a disorder in fractal scaling of human behavior. This possibility could be tested through long-term measurements of human behavior in patients with diabetes and their evaluation by statistical fractal indices determined by the power-law residence-time distribution, cross-correlation coefficient ($\rho^{{\left( {1,2} \right)}} \left( s \right)$), and long-range cross-correlation ($H_{c1}$) (which, in the current study, were found to be regulated by insulin signaling). In parallel, statistical fractal indices obtained from behavioral dynamics in healthy individuals and patients with diabetes can be evaluated by a theoretical NN model representing the human brain structure^43^. Model analyses would provide additional multifaceted connections of multiple properties in behavioral fractal kinetics with brain neural activity. Together, the combination of long-term measurements, fractal statistical analysis, and theoretical neurodynamic modeling of fractal scaling of human behavior is expected to provide a more integrated clinical view of psychiatric symptoms in human patients with diabetes, which could contribute to the development of new diagnostic indices and the improvement of clinical treatment.

## Methods

### *C. elegans* strains and maintenance

*C. elegans* strain Bristol N2 (wild-type) and temperature-sensitive mutants of genes in insulin signaling, CB1370 *daf-2* (e1370) and CF1038 *daf-16* (mu86) were maintained on Nematode Growth Medium (NGM) agar plate at 15 °C.

### Fabrication of microfluidic device and culture of *C. elegans* in microfluidic device

Two microfluidic devices, WormFloII and WormFloI^5^, were fabricated by combining conventional photolithography and soft lithography methods^44^. For WormFloII, a polydimethylsiloxane (PDMS) chip and a bottom PDMS plate with 1-mm thickness were assembled using the oxygen plasma bonding method. Food bacteria suspension (*E. coli*, OP50 strain with OD = 0.1 in a buffer containing 50 mM NaCl, 15 mM K_2_HPO_4_, 96 mM KH_2_PO_4_, 0.3 mM CaCl_2_, 0.3 mM MgSO_4_, 5 µg cholesterol, 1% Tween80 (Tokyo Chemical Industry Co., Ltd., Japan)) were supplied to the WormFloII, whereas M9 buffer with/without 1 g/L glucose were supplied to the WormFloI to maintain animals for observation at 0.4 ml/h with the Micro Ceram pump (MSP-001, Yamazen Corporation, Japan). Due to the structure of the junctional micro-slit channels in each chamber, an introduced adult animal in the chamber was held and its offspring flowed off just after hatching from the chamber. We confirmed the absence of offspring at the end of recording.

### Observation and quantification of *C. elegans* behavior

Animals at the developmental stage after the last molting and before bearing eggs (“young adult stage”) were picked up and cultured at non-permissive temperature for the *daf-2* and *daf-16* mutant strains, 24 °C, for one day and transferred to the microfluidic device that was soaked in M9 buffer in a 15-cm-diameter glass dish at room temperature. Then, the 15-cm-diameter glass dish containing the microfluidic device was installed in a temperature-controlled aluminum box maintained at 25 °C^5^. Temperature acclimation at the start of recording and later maintenance for 3 days were confirmed by direct temperature monitoring^5^. Animals were observed under blue-light-cut illumination by a macroscope with an apochromat objective lens (1 ×) (Z16 APO, Leica, Germany) and recorded in an H264 compressed movie^5^. Animal swimming activity was measured by counting pixels in a bitmap image, in which animal movement was determined by comparing the image at the previous time frame (Supplemental Video 1)^5^. The movie compression effect on swimming activity was corrected on the activity time series by using the moving average^5^.

### Data analysis

For DMA, the fluctuation function $F\left( s \right)$ is obtained by using the mean square root of the detrended noise round series, defined as

1 $$F\left( s \right) = { }\sqrt {\frac{1}{N}\mathop \sum \limits_{i = 1}^{N} \left( {y\left[ i \right] - \tilde{y}_{s} \left[ i \right]} \right)^{2} } .$$

$F\left( s \right)$ is computed from the DRS $\left\{ {x\left[ i \right]} \right\}_{i = 1}^{N}$ by the following procedure: the DRS $\left\{ {x\left[ i \right]} \right\}$ is integrated after removing its mean value to obtain $\left\{ {y\left[ i \right]} \right\}$. This integrated DRS is filtered by the Savitzky-Golay (SG) filter to estimate the trend round series $\left\{ {\tilde{y}_{s} \left[ i \right]} \right\}$, to obtain the detrended noise round series $\left\{ {y\left[ i \right] - \tilde{y}_{s} \left[ i \right]} \right\}$. Then, the $F\left( s \right)$ vs $s$ plot on the log–log scale is fit with a linear function $y = ax + b$ by the least squares method to estimate the Hurst exponent.

For DMCA, the cross-fluctuation function $F^{{\left( {1,2} \right)}}$ is obtained by determining the root of cross-covariance between the bivariate detrended noise round series, defined as

2 $$F^{{\left( {1,2} \right)}} \left( s \right) = { }\sqrt {\frac{1}{N}\mathop \sum \limits_{i = 1}^{N} \left| {\left( {y^{\left( 1 \right)} \left[ i \right] - \tilde{y}_{s}^{\left( 1 \right)} \left[ i \right]} \right)\left( {y^{\left( 2 \right)} \left[ i \right] - \tilde{y}_{s}^{\left( 2 \right)} \left[ i \right]} \right)} \right|} .$$

$F^{{\left( {1,2} \right)}} \left( s \right)$ is computed from bivariate DRSs $\left\{ {\left( {x^{\left( 1 \right)} \left[ i \right],x^{\left( 2 \right)} \left[ i \right]} \right)} \right\}_{i = 1}^{N}$ by using a procedure analogous to DMA. The linear fit to the $F^{{\left( {1,2} \right)}} \left( s \right)$ versus $s$ plot in the log–log scale can provide an estimate of the cross Hurst exponent. In addition, the multi-scale correlation coefficient $\rho^{{\left( {1,2} \right)}} \left( s \right)$, defined as

3 $$\rho^{{\left( {1,2} \right)}} \left( s \right) = { }\frac{{\mathop \sum \nolimits_{i = 1}^{N} \left( {y^{\left( 1 \right)} \left[ i \right] - \tilde{y}_{s}^{\left( 1 \right)} \left[ i \right]} \right)\left( {y^{\left( 2 \right)} \left[ i \right] - \tilde{y}_{s}^{\left( 2 \right)} \left[ i \right]} \right)}}{{\sqrt {\mathop \sum \nolimits_{i = 1}^{N} \left( {y^{\left( 1 \right)} \left[ i \right] - \tilde{y}_{s}^{\left( 1 \right)} \left[ i \right]} \right)^{2} } \sqrt {\mathop \sum \nolimits_{i = 1}^{N} \left( {y^{\left( 2 \right)} \left[ i \right] - \tilde{y}_{s}^{\left( 2 \right)} \left[ i \right]} \right)^{2} } }}$$

is computed to evaluate the existence of an interaction between bivariate detrended noise round series.

### Statistical analysis

Due to rejection of the normality hypothesis for scaling exponents in DMA and DMCA, and the power-law exponents of the residence-time distribution, the non-parametric Wilcoxon rank sum test was employed for pairwise comparisons between groups. In pairwise comparisons, the Benjamini and Hochberg method for correcting the false discovery rate (FDR) was used to deal with multiple testing problems.

### Preprint

A previous version of this manuscript was published as a preprint^46^.

## Supplementary Information

Supplementary Legends.

Extended Data Fig. 1.

Extended Data Fig. 2.

Extended Data Fig. 3.

Extended Data Fig. 4.

Extended Data Fig. 5.

Extended Data Fig. 6.

Extended Data Fig. 7.

Extended Data Fig. 8.

Extended Data Fig. 9.

Supplementary Video 1.

Supplementary Video 2.

Supplementary Video 3.

Supplementary Video 4.

Supplementary Discussion.

## Data Availability

The *C. elegans* swimming activity time series and movie data reported in this paper are deposited in the Systems Science of Biological Dynamics (SSBD) database^45^, https://doi.org/10.24631/ssbd.repos.2021.11.001.
