# Supplementary material for: Insulin signaling shapes fractal scaling of C. elegans behavior
Source: Sci Rep. 2022 Jun 21;12:10481. doi: 10.1038/s41598-022-13022-6 (PMC9213454; doi:10.1038/s41598-022-13022-6)
Supplement: Supplementary file 1 — Supplementary Legends. [file 41598_2022_13022_MOESM1_ESM.docx]

## Extended Data Figure Legends

Extended Data Fig. 1: Activity time series of episodic behavior of fed *C. elegans*

Swimming activity time series of wild-type (**a-e**), *daf-2* (**h-l**), and *daf-16* (**o-s**) fed animals for 3 days. Red-marked regions are magnified in graphs immediately below (e.g., all of **b** represents red region from **a**; all of **c** represents red region from **b**; etc.). DRSs for active (red) and inactive state (blue) in wild-type (**f**, **g**), *daf-2* (**m**, **n**), and *daf-16* (**t**, **u**) fed animals were obtained from activity time series for 3 days (**a**, **h**, **o**). px: number of pixels where animals moved from previous frame.

Extended Data Fig. 2: Activity time series of episodic behavior of *C. elegans* cultured without food bacteria in WormFloI

Swimming activity time series of starved (**a-e**) and glucose-fed (**h-l**) wild-type animals for 3 days. Red-marked regions are magnified in graphs immediately below (e.g., all of **b** represents red region from **a**; all of **c** represents red region from **b**; etc.). Active (red) and inactive (blue) DRSs in starved (**e**, **f**) and glucose-fed (**k**, **l**) wild-type animals were obtained from above activity time series for 3 days (**a**, **h**). px: number of pixels where animals moved from previous frame.

Extended Data Fig. 3: Power-law residence-time distributions of behavioral states of *C. elegans* cultured without food bacteria in WormFloI

Averaged normalized probability density distributions of residence time for active (red) and inactive states (blue) of starved (**a**, **b**) and glucose-fed (**c**, **d**) wild-type animals among individual animals (grey), in log-log plot. Error bars represent standard deviation. Distributions for inactive state (**b**, **d**) were fit with a linear function in a range between -0.5 and 1.5 on x-axis (black line), whereas distributions for active state (**a**, **c**) were fit with a linear function in a range between -0.5 and 0.8 and were extrapolated to 1.5 on x-axis (black line).

Extended Data Fig. 4: Power-law exponents of residence-time distributions for behavioral states of *C. elegans* cultured with/without food bacteria

Box-swarm plots showing raw values and medians with 25th and 75th percentiles of power-law exponents of residence times for active (**a**) and inactive (**c**) states. Error bars represent standard deviations. FDR-corrected P-values by pairwise Wilcoxon rank sum test for active (**b**) and inactive (**d**) states, with P-values > 0.05 shown in grey.

Extended Data Fig. 5: Long-range auto-/cross-correlations and multiscale cross-correlation coefficient of DRSs in *C. elegans* cultured without food bacteria in WormFloI

Averaged noise function $F\left( s \right)$ of active (red) and inactive (blue) DRSs, and averaged cross-noise function $F^{(1,2)}\left( s \right)$ between active and inactive DRSs (green), among individual animals (grey) were plotted against scale $\left( s \right)$ for starved (**a**, **c**) and glucose-fed (**d**, **f**) wild-type animals. $F\left( s \right)$ vs $\left( s \right)$ plots for inactive DRS (blue) were fit with a linear function from 1.1 and 3.1. $F\left( s \right)$ vs $\left( s \right)$ plots for active DRS (red) and $F^{(1,2)}\left( s \right)$ vs $\left( s \right)$ plots (green) were fit in distinct linear functions between 1.1 and 2.1 and between 2.1 and 3.1. Averaged multiscale cross-correlation coefficient between active and inactive DRSs (MCCC; black) among individual animals (grey) were plotted against scale $\left( s \right)$ for starved (**b**) and glucose-fed (**e**) wild-type animals. Error bars represent standard deviations.

Extended Data Fig. 6: Hurst exponents of DRS in animals cultured with/without food bacteria

Raw values and medians with 25th and 75th percentiles of Hurst exponents of active DRS at shorter round scale (**a**), active DRS at longer round scale (**c**), and inactive DRS (**e**). Raw values and medians with 25th and 75th percentiles of Hurst exponents of a cross-correlated component between active and inactive DRSs at shorter round scale (**g**) and longer round scale (**i**), obtained from Fig. 2 and Extended Data Fig. 5, are shown in Box-swarm plot. FDR-corrected P-values from pairwise Wilcoxon rank sum test are shown in the corresponding combinations (**b**, **d**, **f**, **h**, **j**). P-values > 0.05 are shown in grey.

Extended Data Fig. 7: Cross-correlation coefficient between active and inactive DRSs at $\boldsymbol{log}_{\boldsymbol{10}}\boldsymbol{(s)=2.5}$ in animals cultured with/without food bacteria

(**a**) Box-swarm plots showing raw values and medians with 25th and 75th percentiles of cross-correlation coefficients between active and inactive DRSs at ${log}_{10}(s)=2.5$. (**b**) FDR-corrected P-values by pairwise Wilcoxon rank sum test, with P-values > 0.05 shown in grey.

Extended Data Fig. 8: Stepwise computation of DMA and DMCA at longer round scale and the relation with activity time series

(**a**, **h**, **o**) Active (red) and inactive (blue) DRSs in fed wild-type (a), fed *daf-2* (h), and fed *daf-16* animals (o) at longer round scale (3,000 rounds). (**b-c**, **i-j**, **p-q**) Integrated DRSs, obtained by removing average durations of active (solid line; b, i, p) and inactive (solid line; c, j, q) DRSs. S-G filter was fit to integrated active and inactive DRSs, to obtain trend of integrated active (dashed line; b, i, p) and inactive (dashed line; c, j, q) DRSs. (**d**, **k**, **r**) Detrended noise round series (dNRS) for active (red) and inactive (blue) DRSs, obtained by removing trend from integrated DRSs. (**e, l, s**) Scatter plots between active dNRS (y-axis) and inactive dNRS (x-axis). (**f**) Activity time series for active and inactive DRSs in fed wild-type animals at longer round scale (3,000 rounds). (**m**, **t**) Activity time series in fed *daf-2* (m) and fed *daf-16* (t) animals, shown in the same length as fed wild-type animals (f) for comparison. (**g, n, u**) Magnification of 1/100th length of activity time series from (f, m, t). Blue arrows in (a) indicate rounds of very long inactive states in inactive DRS, which correspond to a sudden jump in integrated DRS and dNRS (blue arrows in c, d). Red and blue brackets in (h, o) indicate examples of very long active or inactive rounds that appear at high density, which correspond to a high amplitude of dNRS (red and blue brackets in k, r). Red and blue brackets in (f) indicate active and inactive episodes. Blue arrows in (g) indicate examples of short inactive states within an active episode. px: number of pixels where animals moved from previous frame.

Extended Data Fig. 9: Stepwise computation of DMA and DMCA at shorter round scale and the relation with activity time series

(**a**, **g**, **m**) Active (red) and inactive (blue) DRSs in fed wild-type (a), fed *daf-2* (g), and fed *daf-16* animals (m) at shorter round scale (100 rounds). (**b-c**, **h-i**, **n-o**) Integrated DRSs, obtained by removing average durations of active (solid line; b, h, n) and inactive (solid line; c, i, o) DRSs. S-G filter was fit to integrated active and inactive DRSs, to obtain trend of integrated active (dashed line; b, h, n) and inactive (dashed line; c, i, o) DRSs. (**d**, **j**, **p**) Detrended noise round series (dNRS) for active (red) and inactive (blue) DRSs, obtained by removing the trend from the integrated DRSs. (**e, k, q**) Scatter plots between active dNRS (y-axis) and inactive dNRS (x-axis). (**f**) Activity time series for active and inactive DRSs in fed wild-type animals at shorter round scale (100 rounds). (**l, r**) Activity time series in fed *daf-2* (l) and fed *daf-16* (r) animals, shown in the same length as fed wild-type animals (f) for comparison. Red and blue brackets in DRS (a) indicate alternating appearance of consecutive rounds between longer active states/shorter inactive states (red brackets) and shorter active states/longer inactive states (blue bracket in a). Red and pink brackets in activity time series (f) indicate a time region with high swimming activity (red) or low swimming activity (pink) within a single active episode. Red brackets in (l, r) indicated active episodes. px: number of pixels where animals moved from previous frame.

## Supplementary Figure Legends

Supplementary Video 1: Fed wild-type animals cultured with food bacteria

**(Upper panel)** *C. elegans* swimming behavior in fed wild-type animals. Pixels where animals moved from previous frame are shown in green. **(Lower panel)** Swimming activity (number of green pixels within culture chamber) of animal ID 26 (indicated by red frame in above movie) is highlighted by green line. Vertical dashed line indicates time point shown in frame in above movie. Blue lines at high (active state) and low (inactive state) values indicate activities above or below activity threshold of 12 pixels/frame, respectively. Posing was observed at 8.5, 10, 19.5, 22.5, 15.5, 28.5, 40, 43.5, and 50 sec.

Supplementary Video 2: Fed *daf-2* animals cultured with food bacteria

**(Upper panel)** *C. elegans* swimming behavior in fed *daf-2* animals. Pixels where animals moved from previous frame are shown in green. **(Lower panel)** Swimming activity (number of green pixels within culture chamber) of animal ID 27 (indicated by red frame in above movie) is highlighted by green line. Vertical dashed line indicates time point shown in frame in above movie. Blue lines at high (active state) and low (inactive state) values indicate activities above or below activity threshold of 12 pixels/frame, respectively. Posing was observed at 57 and 58.5 sec.

Supplementary Video 3: Fed *daf-16* animals cultured with food bacteria

**(Upper panel)** *C. elegans* swimming behavior in fed *daf-16* animals. Pixels where animals moved from previous frame are shown in green. **(Lower panel)** Swimming activity (number of green pixels within culture chamber) of animal ID 23 (indicated by red frame in above movie) is highlighted by green line. Vertical dashed line indicates time point shown in frame in above movie. Blue lines at high (active state) and low (inactive state) values indicate activities above or below activity threshold of 12 pixels/frame, respectively. Posing was observed at 8, 15, and 48.5 sec.

Supplementary Video 4: Uniform flow among chambers on WormFloII

M9 buffer containing red food coloring was supplied to the microfluidic device. The weak grey signal that starts to appear in the flow channel at 3 sec is the signal from the red food coloring.
