# Supplementary figures and images for: Insulin signaling shapes fractal scaling of C. elegans behavior
Source: Sci Rep. 2022 Jun 21;12:10481. doi: 10.1038/s41598-022-13022-6 (PMC9213454; doi:10.1038/s41598-022-13022-6)

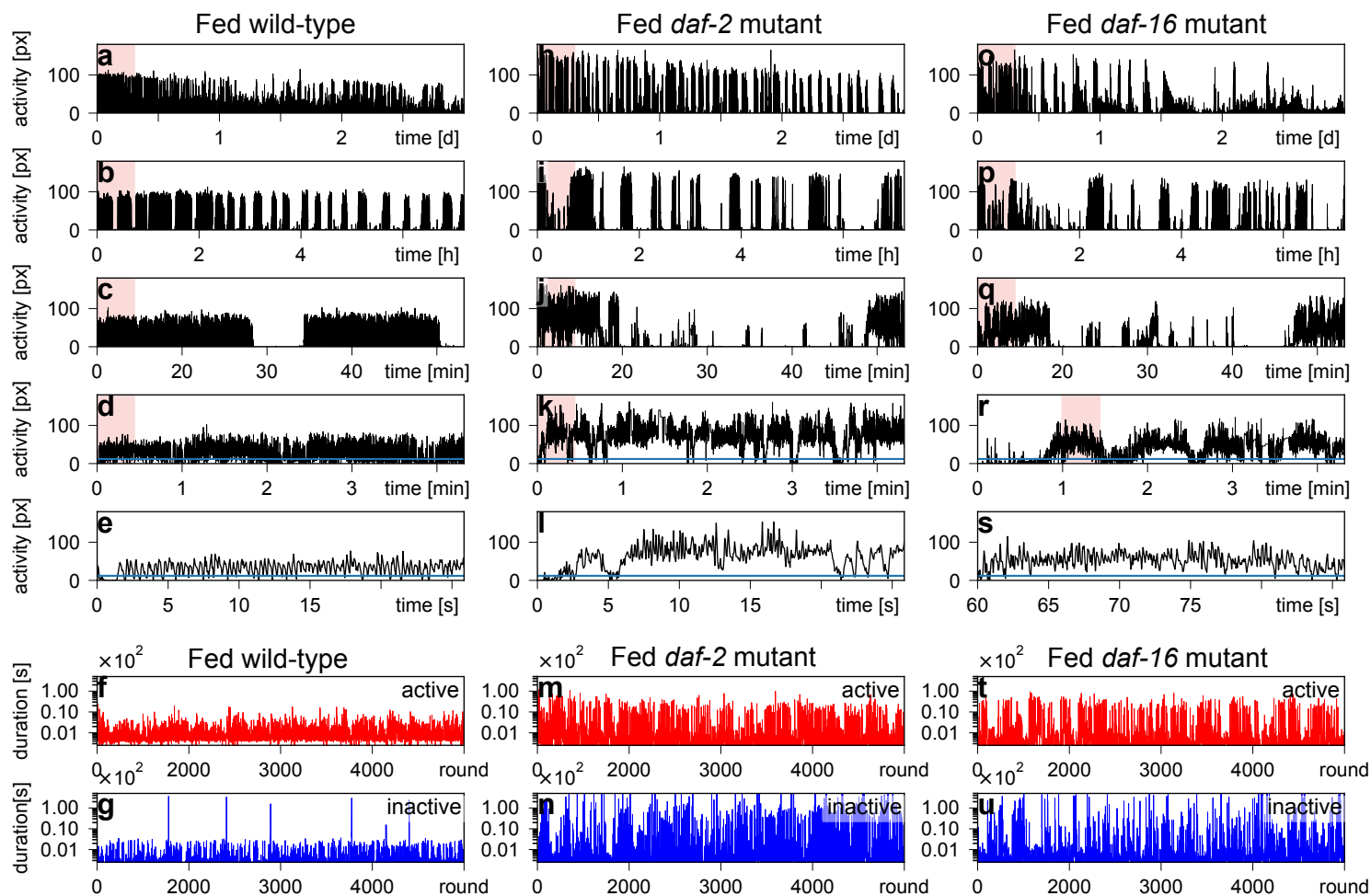

Extended Data Fig 1\_Arata et al

Supplement: Supplementary file 2 — Extended Data Fig. 1. [file 41598_2022_13022_MOESM2_ESM.pdf]

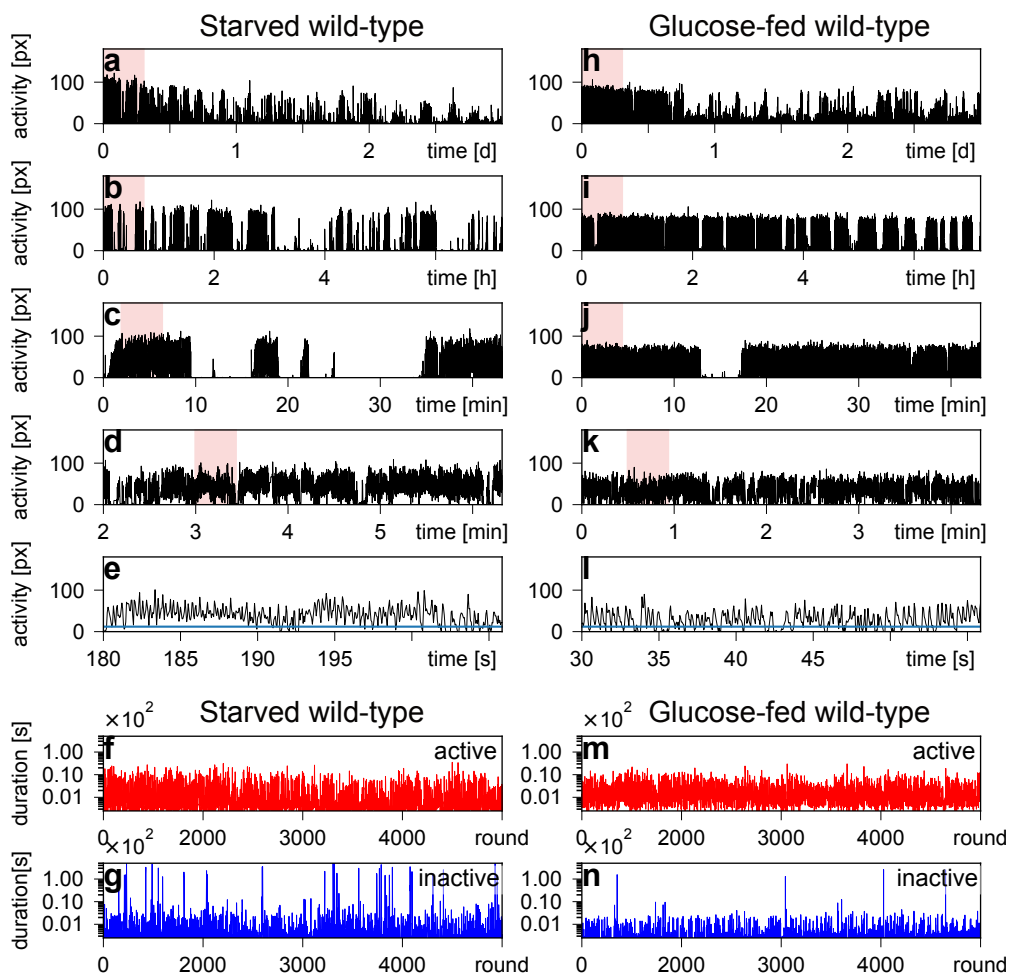

Extended Data Fig. 2\_Arata et al

Supplement: Supplementary file 3 — Extended Data Fig. 2. [file 41598_2022_13022_MOESM3_ESM.pdf]

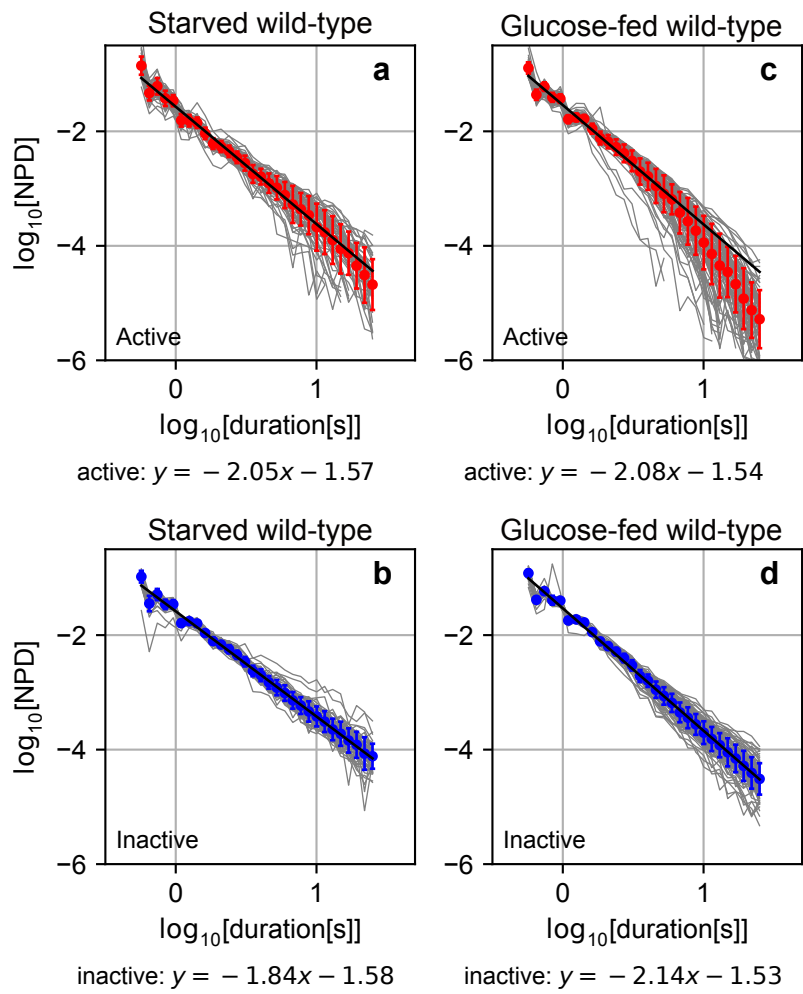

Supplement: Supplementary file 4 — Extended Data Fig. 3. [file 41598_2022_13022_MOESM4_ESM.pdf]

Starved wild-type

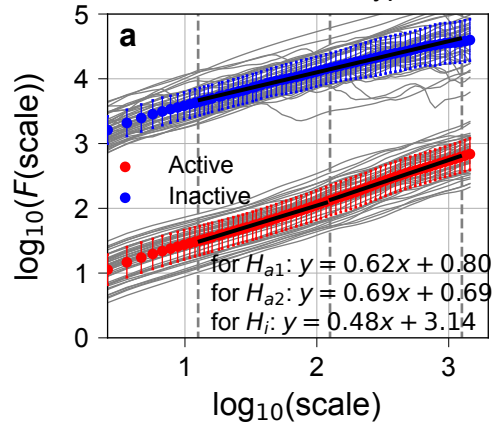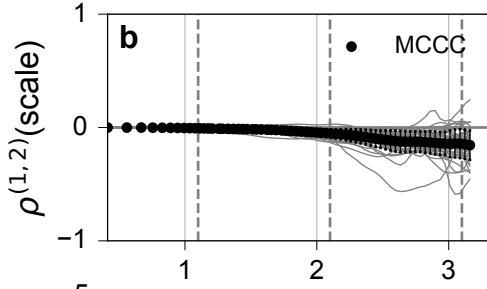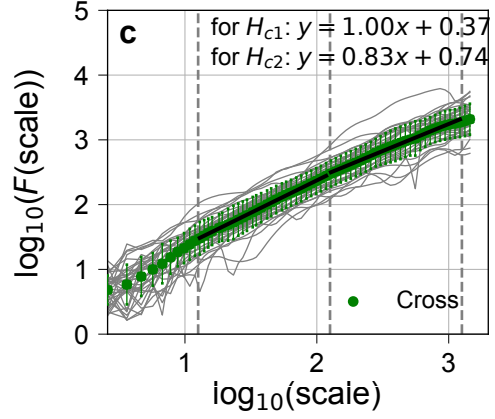

Glucose-fed wild-type

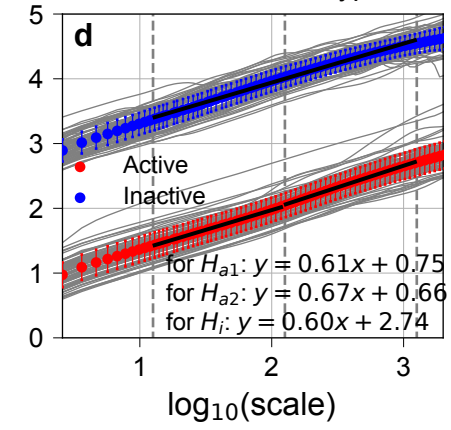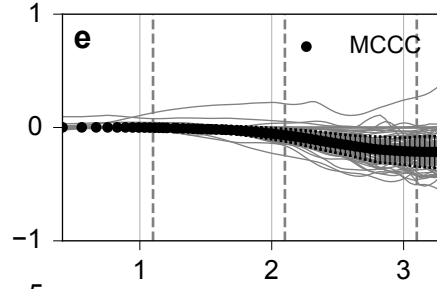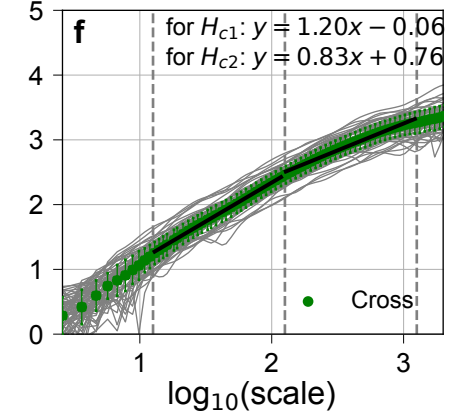

Supplement: Supplementary file 6 — Extended Data Fig. 5. [file 41598_2022_13022_MOESM6_ESM.pdf]

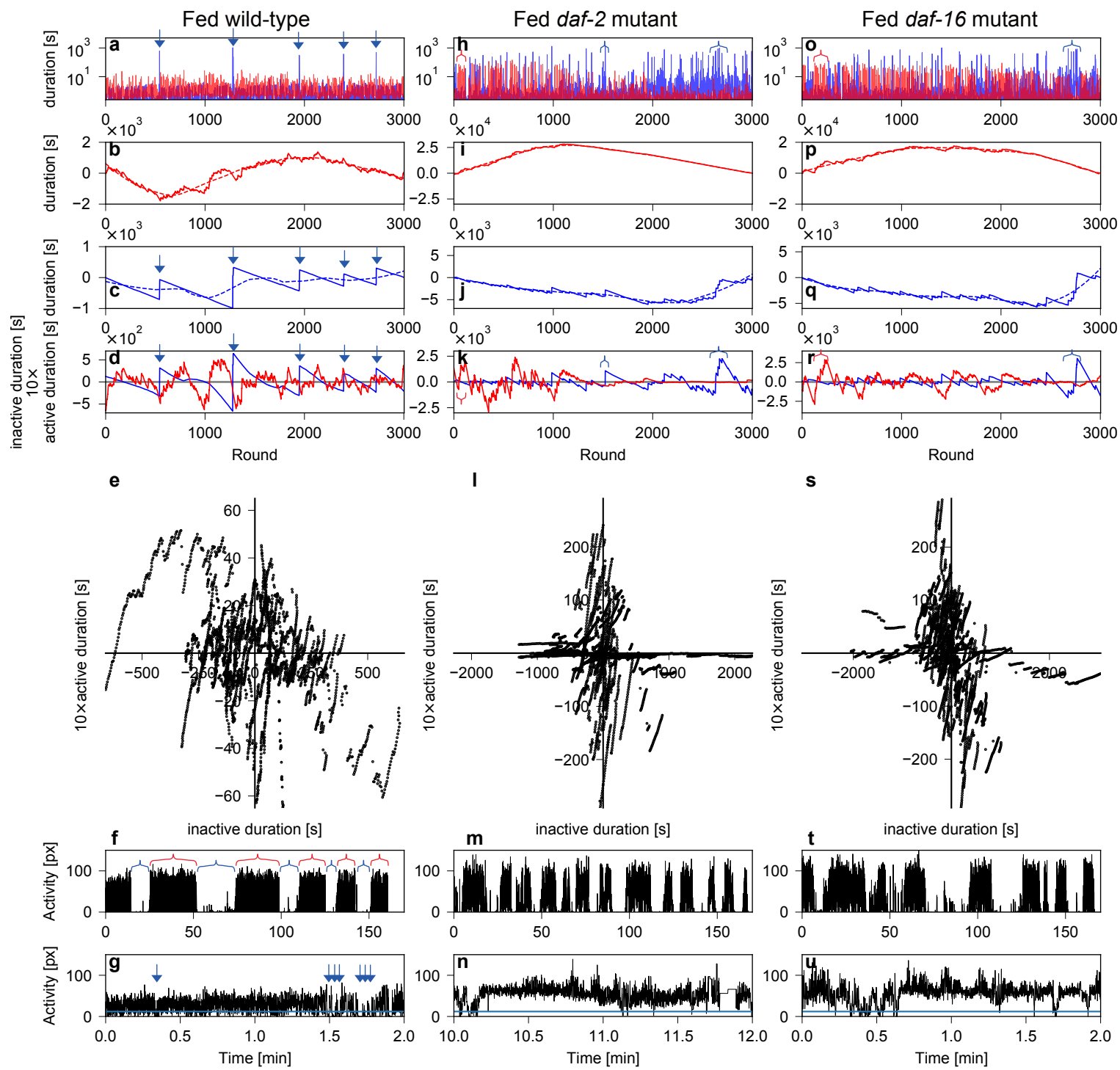

Extended Data Fig. 8\_Arata et al

Supplement: Supplementary file 9 — Extended Data Fig. 8. [file 41598_2022_13022_MOESM9_ESM.pdf]

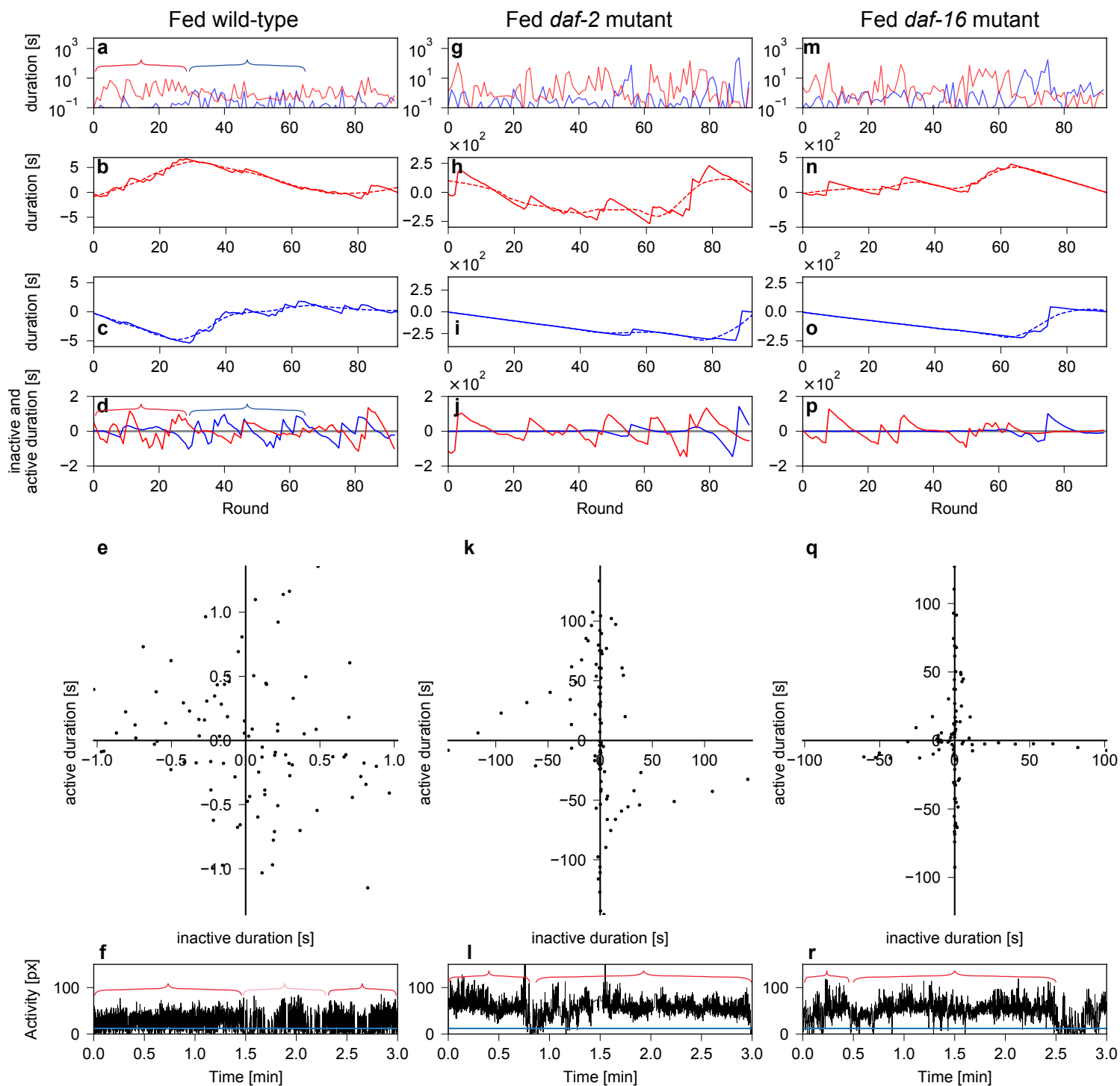

Extended Data Fig. 9\_Arata et al

Supplement: Supplementary file 10 — Extended Data Fig. 9. [file 41598_2022_13022_MOESM10_ESM.pdf]
