# Supplementary material for: Insulin signaling shapes fractal scaling of C. elegans behavior
Source: Sci Rep. 2022 Jun 21;12:10481. doi: 10.1038/s41598-022-13022-6 (PMC9213454; doi:10.1038/s41598-022-13022-6)
Supplement: Supplementary file 5 — Extended Data Fig. 4. [file 41598_2022_13022_MOESM5_ESM.pdf]

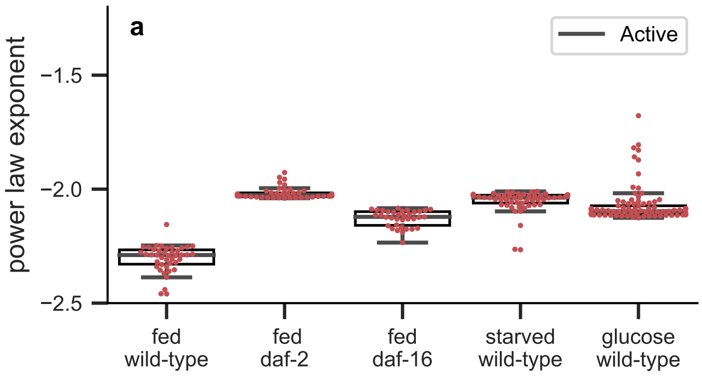

b

	fed wild-type	fed daf-2	fed daf-16	starved wild-type	glucose wild-type
fed wild-type	-	< 0.0001	< 0.0001	< 0.0001	< 0.0001
fed daf-2	-	-	< 0.0001	< 0.0001	< 0.0001
fed daf-16	-	-	-	< 0.0001	< 0.0001
starved wild-type	-	-	-	-	< 0.0001
glucose wild-type	-	-	-	-	-

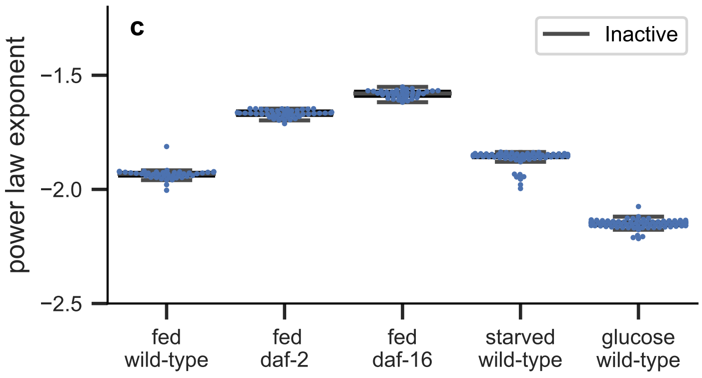

d

	fed wild-type	fed daf-2	fed daf-16	starved wild-type	glucose wild-type
fed wild-type	-	< 0.0001	< 0.0001	< 0.0001	< 0.0001
fed daf-2	-	-	< 0.0001	< 0.0001	< 0.0001
fed daf-16	-	-	-	< 0.0001	< 0.0001
starved wild-type	-	-	-	-	< 0.0001
glucose wild-type	-	-	-	-	-

Extended Data Fig. 4_ Arata et al
