# Supplementary material for: Insulin signaling shapes fractal scaling of C. elegans behavior
Source: Sci Rep. 2022 Jun 21;12:10481. doi: 10.1038/s41598-022-13022-6 (PMC9213454; doi:10.1038/s41598-022-13022-6)
Supplement: Supplementary file 7 — Extended Data Fig. 6. [file 41598_2022_13022_MOESM7_ESM.pdf]

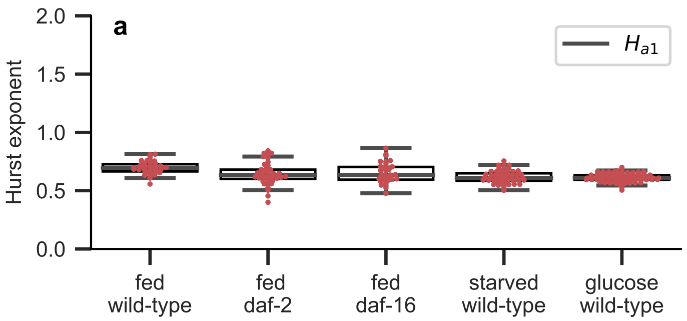

b	fed wild-type	fed daf-2	fed daf-16	starved wild-type	glucose wild-type
fed wild-type	-	0.00035	0.00917	< 0.0001	< 0.0001
fed daf-2	-	-	0.82726	0.139	0.0387
fed daf-16	-	-	-	0.11868	0.04687
starved wild-type	-	-	-	-	0.82726
glucose wild-type	-	-	-	-	-

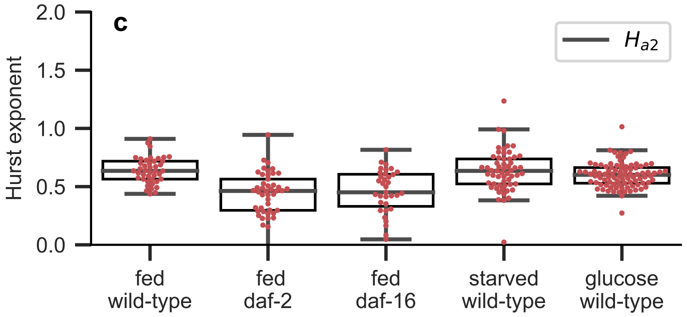

d	fed wild-type	fed daf-2	fed daf-16	starved wild-type	glucose wild-type
fed wild-type	-	< 0.0001	< 0.0001	0.7716	0.05156
fed daf-2	-	-	0.7167	< 0.0001	< 0.0001
fed daf-16	-	-	-	< 0.0001	0.00019
starved wild-type	-	-	-	-	0.17152
glucose wild-type	-	-	-	-	-

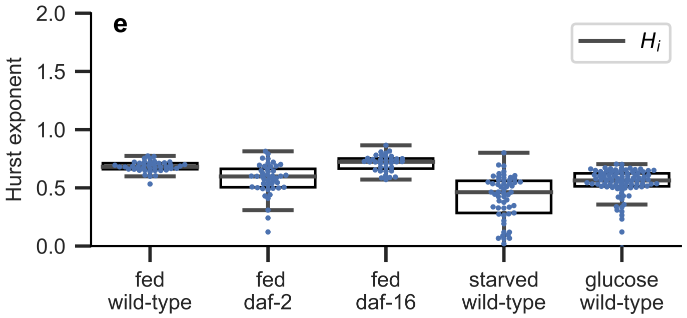

f	fed wild-type	fed daf-2	fed daf-16	starved wild-type	glucose wild-type
fed wild-type	-	< 0.0001	0.0231	< 0.0001	< 0.0001
fed daf-2	-	-	< 0.0001	< 0.0001	0.1741
fed daf-16	-	-	-	< 0.0001	< 0.0001
starved wild-type	-	-	-	-	< 0.0001
glucose wild-type	-	-	-	-	-

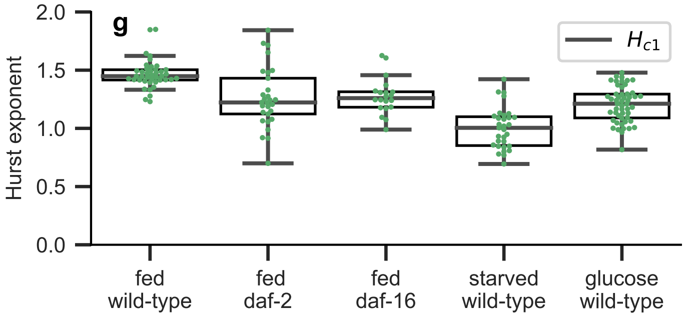

h	fed wild-type	fed daf-2	fed daf-16	starved wild-type	glucose wild-type
fed wild-type	-	< 0.0001	< 0.0001	< 0.0001	< 0.0001
fed daf-2	-	-	0.2366	0.0014	0.867
fed daf-16	-	-	-	< 0.0001	0.1874
starved wild-type	-	-	-	-	< 0.0001
glucose wild-type	-	-	-	-	-

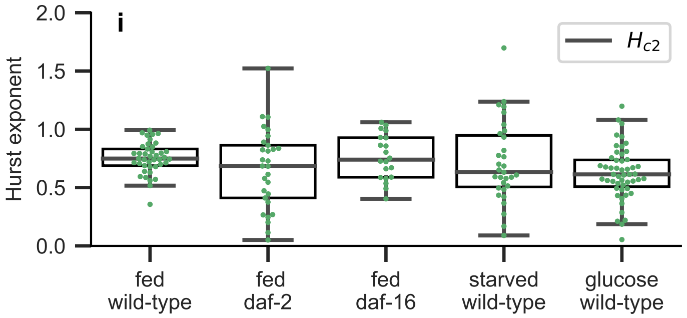

j	fed wild-type	fed daf-2	fed daf-16	starved wild-type	glucose wild-type
fed wild-type	-	0.708	0.708	0.314	0.0023
fed daf-2	-	-	0.7081	0.7081	0.314
fed daf-16	-	-	-	0.529	0.0541
starved wild-type	-	-	-	-	0.565
glucose wild-type	-	-	-	-	-

Extended Data Fig. 6_ Arata et al
