# Supplementary material for: Insulin signaling shapes fractal scaling of C. elegans behavior
Source: Sci Rep. 2022 Jun 21;12:10481. doi: 10.1038/s41598-022-13022-6 (PMC9213454; doi:10.1038/s41598-022-13022-6)
Supplement: Supplementary file 8 — Extended Data Fig. 7. [file 41598_2022_13022_MOESM8_ESM.pdf]

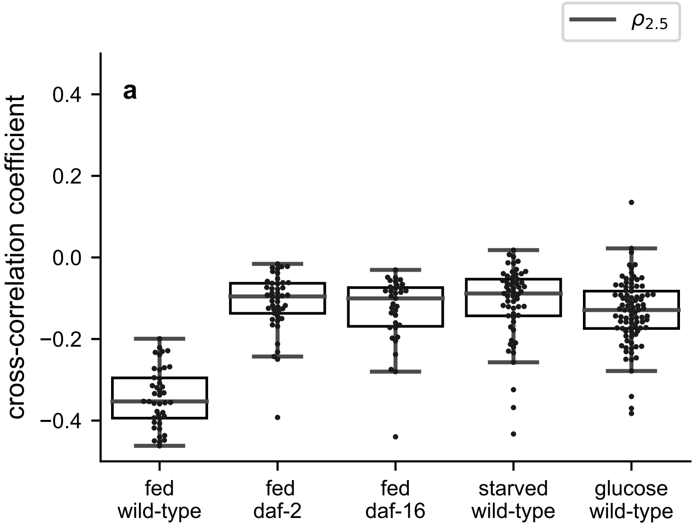

b

	fed wild-type	fed daf-2	fed daf-16	starved wild-type	glucose wild-type
fed wild-type	nan	< 0.0001	< 0.0001	< 0.0001	< 0.0001
fed daf-2	nan	nan	0.411	0.574	0.049
fed daf-16	nan	nan	nan	0.262	0.4107
starved wild-type	nan	nan	nan	nan	0.023
glucose wild-type	nan	nan	nan	nan	-

Extended Data Fig. 7_ Arata et al
